# How much of the predisposition to Hashimoto’s thyroiditis can be explained based on previously reported associations?

**DOI:** 10.1007/s40618-018-0910-4

**Published:** 2018-06-21

**Authors:** A. Jabrocka-Hybel, A. Skalniak, J. Piątkowski, R. Turek-Jabrocka, P. Vyhouskaya, A. Ludwig-Słomczyńska, J. Machlowska, P. Kapusta, M. Małecki, D. Pach, M. Trofimiuk-Müldner, K. Lizis-Kolus, A. Hubalewska-Dydejczyk

**Affiliations:** 1 Department of Endocrinology, Medical Faculty, Jagiellonian University Medical College, Krakow, Poland; 2 Department of Endocrinology, University Hospital in Krakow, ul. Kopernika 17, 31-501 Krakow, Poland; 3 Department of Medical Diagnostics, Pharmacy Faculty, Jagiellonian University Medical College, Krakow, Poland; 4 Center for Medical Genomics OMICRON, Jagiellonian University Medical College, Krakow, Poland; 5 Department of Metabolic Diseases, Medical Faculty, Jagiellonian University Medical College, Krakow, Poland; 6 Endocrinology Department-Oncology Center, Kielce, Poland

**Keywords:** Hashimoto thyroiditis, Genetics, Autoimmune, Polymorphism, HLA

## Abstract

**Purpose:**

Our insight in the genetics of Hashimoto’s thyroiditis (HT) has become clearer through information provided by genome-wide association studies and candidate gene studies, but remains still not fully understood. Our aim was to assess how many different genetic risk variants contribute to the development of HT.

**Methods:**

147 HT cases (10.2% men) and 147 controls (13.6% men) were qualified for the analysis. Intrinsic and environmental factors were controlled for. Polymorphisms (SNP) were chosen based on the literature and included markers of the genes *PTPN22, CTLA4, TG, TPO* among others, and of genomic regions pointed by GWAS studies. SNP were typed on a microarray. Variants in the *HLA*-*DRB1* gene were identified by Sanger sequencing.

**Results:**

Multivariate predisposition to HT was modeled. Based on the investigated group, a model of seven variables was obtained. The variability explained by this model was assessed at only 5.4821% (*p* = 2 × 10^−6^), which indicates that many dozens of factors are required simultaneously to explain HT predisposition.

**Conclusions:**

We analyzed genetic regions commonly and most significantly associated with autoimmune thyroid disorders in the literature, on a carefully selected cohort. Our results indicated a lack of possibility to predict the risk of HT development, even with a multivariate model. We therefore conclude that strong associations of single genetic regions with HT should be interpreted with great caution. We believe that a change in the attitude towards genetic association analyses of HT predisposition is necessary. Studies including multiple factors simultaneously are needed to unravel the intricacies of genetic associations with HT.

**Electronic supplementary material:**

The online version of this article (10.1007/s40618-018-0910-4) contains supplementary material, which is available to authorized users.

## Introduction

Autoimmune responses target the thyroid more frequently than any other organ. The term autoimmune thyroid disease (AITD) refers to the whole spectrum of thyroid autoimmunity ranging from subclinical disease manifesting by the presence of autoantibodies and only slightly elevated TSH levels to clinical disease with typical symptoms, high TSH and low FT4 and FT3. The prevalence of autoimmune thyroid disorders such as Graves’ disease (GD) and Hashimoto’s thyroiditis (HT) is estimated to be 5% in the overall population [1, 2], but the prevalence of subclinical disease manifesting by the production of anti-thyroid antibodies without clinical disease is even higher [3]. Women are affected 4–5 times more often than men [3, 4].

HT, also called chronic lymphocytic or autoimmune thyroiditis, is part of the spectrum of chronic autoimmune thyroid diseases and is associated with various degrees of thyroid hypofunction, with lymphocytic infiltration and with thyroid autoantibodies production. Thyroid peroxidase (TPO) and thyroglobulin (Tg) are the major autoantigens in Hashimoto’s disease, but antibodies against those antigens (TPO-Ab and Tg-Ab) occur also in ~ 70% of patients with GD. The thyroid-stimulating hormone receptor (TSHR) is the major autoantigen in Graves’ disease, but TSHR antibodies may occur also in some patients with Hashimoto’s disease [5]. Autoimmune thyroiditis can coexist with other endocrine and non-endocrine autoimmune disorders. Autoimmune polyglandular syndromes (APSs) show a great heterogeneity of syndromes and manifest sequentially with a large time interval between the occurrence of the first and the second glandular autoimmune disease [6]. For example, the term “thyrogastric syndrome” defines the association between AITD and chronic autoimmune gastritis (CAG), and was first described in the early 1960s. More recently, this association has been included in polyglandular autoimmune syndrome type IIIb, in which autoimmune thyroiditis represents the pivotal disorder [7, 8].

The main known susceptibility genes for AITD can be divided into thyroid-specific genes and immunoregulatory genes [5]. Identified thyroid-specific genes are *TSHR* and *Tg*, with the *TPO* gene being of uncertain significance. Single-nucleotide polymorphisms (SNPs) in *TSHR* have been specifically associated with Graves’ disease (but not HT) in the Caucasian population [9, 10].

*TG* is one of the most important candidate genes for AITD, since thyroglobulin accounts for about 80% of the total thyroid protein content. Even though Tg antibodies are not the most sensitive or specific for diagnosing autoimmune thyroiditis, Tg has been recognized as a potential early trigger of autoimmune thyroiditis. Supporting a key role for Tg in triggering AITD are data showing that *TG* is a major AITD susceptibility gene [11–14].

Apoptosis is proposed as one of the mechanisms which could initiate Hashimoto’s thyroiditis. The Fas/Fas ligand cascade represents a major pathway-inducing apoptosis. Erdogan et al. showed that a gene polymorphism of Fas may play a role in the regulation of apoptosis in thyroid autoimmune disorders [15].

Variants in immune-modulating genes (such as *FOXP3, CD25, CD40, CTLA4,* and *HLA*) have also been recognized as risk factors for different autoimmune disorders in case–control studies. Polymorphisms in these genes are not specific for AITD, as they also confer susceptibility to other autoimmune diseases. In a recent study by Houcken et al., *PTPN22* and *CTLA4* polymorphisms have been shown to be associated with APS and differentiate between polyglandular and monoglandular autoimmunity. The *PTPN22* c.1858C>T (rs2476601) allele and genotype distribution were markedly different between APS, type 1 diabetes, Graves’ disease and controls. In addition, SNP *CTLA4* CT60 (rs3087243) G-allele carriers were more frequently observed in the APS (85%) than in the control group (78%) [16].

A lot of work has been published by different authors who aimed to correlate HT with the presence of variants in single genes. Since a couple of years, it seems, however, clear that at the level of SNP association studies we are not able to identify single genes which would be crucial for the presence of the disease, and researchers tend to the theory that each among several genes might have only a small partial effect on the disease. In the past years, the complexity of genetic factors that influences the development of autoimmunity has become increasingly clear through genetic information provided by both genome-wide association studies (GWAS) and high throughput sequencing. The aim is to understand how genetic risk variants contribute to the development of the disease. It seems that only the simultaneous presence of various genetic and environmental factors altogether may overcome a predisposition threshold and lead to the clinical outcome of HT [17, 18]. Genetic variants provide the primary risk for AITD. When this primary genetic risk interacts with environmental factors (e.g. infection, diet, iodine exposure), the synergistic effect can trigger the disease (e.g. [19]).

In this paper, we aimed to combine multiple genetic regions in one study on a carefully selected cohort, and to obtain a multivariate model which would allow to assess how much of the predisposition to HT can be explained by the simultaneous presence of predisposing variants in more than one gene. This might also allow to assess the number of factors which should be investigated in patients to obtain a model which would explain the majority of the disease predisposition.

## Materials and methods

### Compliance with ethical standards

The study was approved by the Bioethical Committee of the Jagiellonian University in Krakow (No. KBET/68/B/2011). All patients were informed about the reasons of the investigation and gave their written informed consent prior to participation in the study.

### Patients

All participants of the study were of Polish origin. Hashimoto’s thyroiditis was diagnosed by documented primary hypothyroidism requiring thyroid hormone replacement, and the presence of autoantibodies to thyroid peroxidase and/or anti-Tg antibodies, with or without goiter.

- Inclusion criteria for the HT group: TSH above 4.5 µIU/ml, the presence of anti-TG and/or anti-TPO antibodies, typical for HT ultrasound of the thyroid.
- Inclusion criteria for controls: the absence of any inclusion criterion for HT, negative history for thyroid diseases among participants and their first degree relatives.
- Exclusion criteria: other thyroid disorders (thyroid nodular and non-nodular goiter, Graves’ disease, thyroid inflammation), increased anti-TPO antibodies with normal TSH levels (only patients with elevated TSH values were qualified for the study to obtain a homogeneous group for analysis with full disease characteristics), increased antibodies against TSH receptor, lack of consent to participate in the study.

None of the participants were related to each other. 147 HT cases (10.2% men) and 147 controls (13.6% men) were qualified for the analysis. These groups were matched for gender (*p* = 0.4717), age (*p* = 0.9923), marital status (*p* = 0.8148), education (*p* = 0.1157), monthly income (*p* = 0.7793), and size of the city they live in (*p* = 0.8535).

### Demographic data and environmental factors

Non-genetic factors which might play a role in thyroid autoimmunity were assessed by an environmental survey. Patients answered questions regarding demographic data including gender, age, marital status, completed education level, their monthly gross income per person and size of city they live in (as a simple measure of air pollution). Questions about environmental factors included tobacco smoking, alcohol consumption, stress, and a dietary questionnaire. Based on the dietary questionnaire, the individual monthly iodine, selenium and fiber intake was calculated.

### DNA isolation, quantification and quality control

DNA was isolated from whole blood samples collected on EDTA, with the NucleoSpin Blood kit (Macherey–Nagel), according to the Manufacturer’s protocol. The amount and quality of DNA were assessed on NanoDrop 2000 (Thermo Fisher Scientific).

### Genetic studies

Our study included genomic regions most commonly mentioned in connection with AITD in the earlier literature, independently on the effect size obtained by the authors. The regions under investigation included the genes *PTPN22, CTLA4, HLA*-*DRB1, IL10, IL6, TPO, IFIH1, TNF, TG, ZFAT, RET, ARID5B, CD40, FOXP3*, promoter regions, as well as regions typed out for AITD by GWAS on chromosomes 12 (gene *DCN*), 13 (*ARGLU1, FAM155A*), 14 (*TTC9, MAP3K9, RGS6, PSEN1*), 20 (*MAFB, PLCG1, CHD6*), and X (*TNMD, SYTL4, NOX1*) [12, 20]. The gene *IFIH1* was typed out by our team as an additional candidate gene based on the literature, although not commonly investigated so far in AITD. Analyzed polymorphisms are listed in supplementary Table 1. Polymorphisms (SNP) in the above-mentioned regions were investigated on Illumina HiScan using Illumina’s Golden Gate custom panel, according to the Manufacturer’s protocol.

**Table 1:** Tested environmental factors and *p* values of the appropriate statistical tests as stated in the section Methods

	HT group	Ctrl group	*p* value
Tobacco smoking status—whenever during life (yes/no)			
Yes	30.6% (*n* = 45)	31.3% (*n* = 46)	
No	63.3% (*n* = 93)	60.5% (*n* = 89)	0.7682
Pack-years of tobacco smoking—median value and interquartile range			
	2.5 (0.6–6.0)	4.7 (0.7–9.0)	0.4601
Amount of consumed alcohol (g pure ethanol/month)—median value and interquartile range			
	30 (20–50)	40 (20–60)	0.0728
Amount of stressful life situations			
Hard to tell	10.9% (*n* = 16)	4.8% (*n* = 7)	0.5124
Less than once/month	2.0% (*n* = 3)	0.7% (*n* = 1)	
Once/month	1.4% (*n* = 2)	1.4% (*n* = 2)	
Several times/month	8.8% (*n* = 13)	14.3% (*n* = 21)	
Once/week	4.8% (*n* = 7)	8.2% (*n* = 12)	
Several times/week	36.1% (*n* = 53)	42.9% (*n* = 63)	
Every day	15.0% (*n* = 22)	8.8% (*n* = 13)	
Several times/day	19.7% (*n* = 29)	17.0% (*n* = 25)	
Iodine intake (µg/day)—median value and interquartile range			
	272.4 (236.8–324.1)	271.6 (231.2–323.4)	0.7631
Selenium intake (µg/day)—median value and interquartile range			
	116.8 (83.9–162.8)	121.3 (89.9–157.8)	0.7008
Fiber intake (g/day)—median value and interquartile range			
	38.6 (30.3–51.6)	39.5 (30.5–51.1)	0.6773

Variants in the *HLA*-*DRB1* gene were identified by Sanger sequencing of exon 2 of the gene, which includes most of the gene’s heterogeneity. PCR was performed with FirePol polymerase (Solis BioDyne), according to the Manufacturer’s recommendations, on a BioRad T100 thermocycler. 120 ng DNA were used in each 25 µl reaction. Group-specific primers and PCR conditions were used according to van Dijk et al. [21]. Products were visualized electrophoretically, and the remaining reaction mixture purified with NucleoSpin 96 PCR Clean-up (Macherey–Nagel). The products were eluted with 70 µl nuclease-free water (Ambion), dried for 2 h at 75 °C, and pellets were resuspended in 11 µl nuclease-free water. The 10 µl sequencing PCR mixture was performed with BigDye Terminator v3.1 (ThermoFisher Applied Biosystems) and 5× sequencing buffer, 0.7 µl of the appropriate 10 µM sequencing primer (according to [21]), and 5.3 µl of the purified PCR product. The sequencing conditions were in agreement with the Manufacturer’s recommendations, with an annealing temperature of 55 °C. Products were purified by ethanol precipitation, and pellets resuspended in 20 µl nuclease-free water for capillary electrophoresis (ABI 3500, Applied Biosystems).

### Statistical analyses

Statistical analyses were performed in Statistica v12.

#### Demographic data and environmental factors

For categorical data, the Chi-squared test was applied to compare groups. For quantitative and ordinal data, the Mann–Whitney test with correction for incontinuity was used, with an additional control for tied ranks in case of ordinal scale of the data. Results were considered significant at *α* = 0.05.

#### HLA-DRB1

Sequencing results were visualized with DNA Baser v4 and aligned to the NCBI dbMHC database with use of the SBT interface. Association analysis of serotypes and alleles was performed with Statistica v12. Alleles were re-coded into amino acid sequences to visualize non-synonymous regions.

The CHI^2 test was used for association analysis of alleles. Associations were considered significant at *α* = 0.05. Logistic regression was used to reveal a potential pattern of the binding cleft, as has been suggested previously [22].

#### Polymorphism genotyping

Quality control was performed with the Genotyping module of BeadStudio, and based on the GenCall validation. Initial laboratory quality assurance (QA) relied on the GenCall score, a quality metric indicating the reliability of called genotypes that is generated by the BeadStudio software. For initial QA, a sample and SNP GenCall_p10 score threshold of 0.38 and call rate threshold of 85% were set. All analyzed polymorphisms met those criteria. Data were further analyzed with the tool Plink v1.9 [23]. All SNPs and all patients had less than 10% missingness. All polymorphisms under investigation were confirmed to meet the Hardy–Weinberg equilibrium in the control group (*α* = 0.05). Association of each SNP with HT was determined by Cochran–Armitage trend test (Results are shown in supplementary Table 1). The most probable mode of inheritance for each SNP was examined using the permutation procedure implemented in Plink. All polymorphisms were included in the further stepwise regression analysis, independently of whether they appeared to be significantly associated with HT in the standard association analysis.

#### Genetic model

Stepwise logistic regression was performed to obtain a model of genetic regions significantly implicated in the predisposition to HT in our sample group. All polymorphisms in study were included in the analysis, considering their mode of inheritance. Also included was phenylalanine at position 67 of the *HLA*-*DRB1* gene, and the intrinsic factors sex and age. Logistic regression has been chosen because genetic as well as non-genetic factors were considered simultaneously. This method also allows to automatically exclude redundant variables, which is a problem in case of linkage disequilibrium. The goodness of fit for the model was assessed by tenfold cross-validation. The explained predisposition to HT based on the obtained model was assessed with the program GCTA [24], setting the frequency of HT at 1% [25].

## Results

### Environmental factors

The HT and control groups were tested for environmental factors which are known or suggested to play a role in thyroid autoimmunity. Those factors are listed in Table 1.

The HT and control groups did not differ in means of the investigated environmental factors.

#### HLA-DRB1

The percentages of different HLA-DRB alleles were in agreement with frequencies stated at http://www.allelefrequencies.net for the Polish population (*n* = 20,653 alleles) (data not shown).

Stepwise logistic regression analysis which included only amino acid positions suggested by Menconi et al. [22], revealed a weak correlation between Tyr-30 and Arg-71 with HT. The only allele significantly associated with HT in our study was *HLA*-DRB1*11:04, but only as long as no correction for multiple comparisons was applied (*p* = 0.0381). This allele is characterized by the presence of Tyr-30 and Arg-71. Arginine at position 71 has, however, been previously shown by Menconi et al. to play a protective role [22].

If all non-synonymous amino acid positions were analyzed, only phenylalanine at position 67 turned out to be significantly associated with HT in our study group (*p* = 0.0060, OR = 1.675, 95% CI 1.161–2.417).

#### Genotyping results

Among 37 typed polymorphisms, 8 (21.6%) were significantly associated with HT in the single variant association analysis at *α* = 0.05. Those were *PTPN22* rs3811021 (*p* = 0.0333), *PTPN22* rs2476599 (*p* = 0.0027), *TPO* promoter region rs11211645 (*p* = 0.0127), *IFIH1* rs1990760 (*p* = 0.0281), intergenic region at chr. 13q33.3 (*p* = 0.0449), *RGS6* rs2074953 (*p* = 0.0047), *TNMD* rs5966709 (*p* = 0.0023), and *NOX1* rs5921679 (*p* = 0.0375).

However, after Bonferroni correction, none of the polymorphisms itself was significantly associated with HT.

Results of single variant association analyses for all tested SNPs are presented in Supplementary Table 1.

### Stepwise logistic regression model

The multivariate predisposition to HT was modeled. Based on the investigated group, a model of 7 variables was obtained, which included polymorphisms in the genes *HLA*-*DRB1* on chromosome 6, *PTPN22* on chr. 1, *NOX1* on chr. X, a polymorphism downstream from *FAM155A* on chr. 13, *IFIH1* on chr. 2, *RGS6* on chr. 14 and *TNMD* on chr. X, all under the dominant inheritance model. The multivariate model is summarized in Table 2.

**Table 2:** Multivariate HT predisposition model

Variable	Gene or region	*p*	OR (95% CI)
Intercept	–	0.2244	0.617 (0.283–1.345)
rs17886918_T	*HLA*-*DRB1,* Phe-67	0.0217*	1.889 (1.097–3.253)
rs3811021_G	*PTPN22*, untranslated region	0.0200*	1.946 (1.110–3.409)
rs5921679_A	*NOX1*, intronic variant	0.0007*	2.593 (1.495–4.498)
rs2769917_A	3′ from *FAM155A*	0.0210*	2.647 (1.158–6.053)
rs3747517_A	*IFIH1*, His843Arg	0.0117*	2.043 (1.172–3.560)
rs2074953_G	*RGS6*, intronic variant	0.0053*	0.462 (0.269–0.795)
rs5966709_A	*TNMD*, intronic variant	0.0077*	0.454 (0.254–0.811)

Associations were considered significant at *α* = 0.05

*p* value from logistic regression, *OR* odds ratio, *CI* confidence interval

Regression fitting parameters for the model were as follows. Wald’s test for the model: *p* = 3 × 10^−6^, Hosmer–Lemeshow test: *p* = 0.9490. AUC values were 0.730 ± 0.029 and 0.688 ± 0.031 for the basic training dataset and tenfold cross-validation, respectively.

The variability explained by the obtained model was assessed at 5.4821% (*p* = 2 × 10^−6^), which can be interpreted in that almost 5.5% of the predisposition to HT can be explained by the obtained model in the investigated group of patients and cases.

The predictive ability of the model was 65.3 and 64.6% for affected and unaffected, respectively.

## Discussion

In their work, Menconi et al. typed amino acids Tyr-26, Tyr-30, Gln-70, Lys-71 and Arg-74 as strongly associated with HT, with the strongest effect observed for Lys-71 [22]. Such a pattern is present in the allele *HLA*-DRB1*03:01, which was the only significantly associated allele in their study. In our study, no such correlation could be confirmed. The only significant association for *HLA*-*DRB1* found in our study concerned position 67 with phenylalanine. Other hydrophobic and non-polar amino acids (leucine, isoleucine) at this position have been previously correlated with rheumatoid arthritis [26, 27], and amino acid region 67–74 is the most commonly associated with this and other autoimmune disorders [28].

According to Menconi et al. [22], arginine at position 71 should have a strong protective effect, while in our study this amino acid was present in the only HT-associated allele *HLA*-DRB1*11:04. It should be noted that both studies, the one of Menconi et al. [22], as well as our own, are based on very limited numbers of investigated patients and controls: in the cited paper 94 patients and 153 controls were studied. In our study, we analyzed 147 cases and 147 controls. For a region as heterogeneous as the genomic *locus* which includes the gene *HLA*-*DRB1*, accidental correlations are very likely to be obtained in small sample groups. The small sample number combined with slight differences in the recruitment parameters in both studies might have generated the observed discrepancies.

We obtained a model of seven variables (genomic regions) that have the greatest combined effect on HT presence in our study group. *HLA*-*DRB1* and *PTPN22* are genes involved in the immune response, which have been commonly associated with HT and other autoimmune disorders in the literature. Houcken et al. showed that the genotype distribution of a *PTPN22* polymorphism is markedly different between APS, type 1 diabetes, Graves’ disease and controls [16]. *IFIH1* is another immune-regulatory gene, which has been included in our study as a new candidate gene based on literature. The remaining SNPs in the model are markers of regions which have been typed by GWAS in previously published papers. While rs2769917 is a marker of a region correlated with HT, rs5921679 (*NOX1*), rs2074953 (*RGS6*) and rs5966709 (*TNMD*) are located in regions which have been previously correlated specifically with GD, but not with HT [12, 20].

Of course, the number of variables is limited by the number of study participants. It seems probable that the polymorphisms included in the model, or even the genes in the model, would differ if another population had been investigated. It can also not be ruled out that non-genetic factors or gene–gene or gene–environment interactions may be necessarily considered in a genetic model to improve its parameters. In our study, environmental factors have been controlled for before modelling, and thus did not appear significant enough to become included in the model.

Independently of the above considerations, we were able to type 7 most important polymorphisms for HT, in well-defined groups of cases and controls. Each of these genetic regions has been claimed in the literature to be of significant importance for HT development, and no other better susceptibility markers for this disease have been identified so far. Still, their combined effect accounts for only a very small percentage of the observed variance. The obtained seven-variable model was able to explain less than 5.5% of the study group variance (i.e. HT predisposition). This seems quite reliable when compared to about 20% variance explained by a 27-loci model for vitiligo, published by Jin et al. [29]. It, however, also means that a good predictive HT model should include dozens or even hundreds of different parameters. This, in turn, is possible only with extremely large study groups.

We want to emphasize in this paper that each single investigation on single genetic regions in case of diseases of complex etiology should be interpreted with great caution. Pathogenically, multifactorial diseases such as HT require complex analyses during genetic testing. We think, at the present state, that there is little need to undertake new association studies on single well-documented genes which have been previously investigated extensively. Based on our results, we expect that at least a few dozen genetic regions affect the predisposition to HT. Testing an appropriately large group would reveal many more variables and might allow to generate a model which would apply to new patients. We therefore believe that further research should be undertaken that focus not only on single genetic factors, but also on the impact of the simultaneous presence of different genetic variants and environmental factors, and possibly also interactions between them. Unfortunately, so far, insufficient attention has usually been paid to environmental and intrinsic factors in genetic studies on HT predisposition, while differences between the tested and control group may impact genetic results in an unpredictable way.

Our results also stress how different results can be obtained depending on inclusion and exclusion criteria used in different studies. For example, there is still no agreement of whether patients with positive thyroid autoantibodies but no clinical symptoms should be included in the study as the case group or excluded from further analysis. The decision of the investigators regarding this issue may, however, have an impact on the obtained results. This can be clearly seen on the example of *HLA*-*DRB* discussed above, but also in the fact that some of the significantly associated regions in our model have previously been shown by others to be specifically associated only with GD. HT groups are usually more heterogeneous than GD groups, thus specific effects are supposed to be weaker than for GD patients. It therefore seems more probable to oversee significant but relatively weak effects for HT, especially when there is a clinically heterogeneous population under investigation. All this makes it difficult to compare results of different studies, and also to perform literature-based meta-analyses.

Our final model included only genetic factors, while a number of environmental factors were controlled for during recruitment of participants. It has previously been shown that environmental factors as well play a significant role for AITD risk [30, 31]. Those factors still remain mainly unknown, it should, however, be considered in future studies to include environmental parameters in analyses, or at least try to control known environmental factors, when working on HT predisposition models.

## Conclusions

Hashimoto’s thyroiditis (as probably most autoimmune disorders) is a disease with complex background, with dozens or hundreds of different factors influencing the risk of each individual case. These factors might differ between individuals. To obtain a good predictive model, studies on this topic should focus on the simultaneous analysis of multiple genetic and environmental factors.

## Electronic supplementary material

Below is the link to the electronic supplementary material.

Supplementary material 1 (PDF 72 kb)
