# Supplementary material for: How much of the predisposition to Hashimoto’s thyroiditis can be explained based on previously reported associations?
Source: J Endocrinol Invest. 2018 Jun 21;41(12):1409–16. doi: 10.1007/s40618-018-0910-4 (PMC6244553; doi:10.1007/s40618-018-0910-4)
Supplement: Supplementary file 1 — Supplementary material 1 (PDF 72 kb) [file 40618_2018_910_MOESM1_ESM.pdf]

Supplementary table 1

Genotyping results for genomic regions previously associated with HT in the literature (chosen in 2013). Results of single variant association analyses are presented. After Bonferroni correction, none of the polymorphisms itself is significantly associated with HT at the corrected α=0.00135.
CHR, chromosome location according to human reference genome Assembly GRCh38/hg38; SNP, polymorphism signature according to NCBI dbSNP; ALLELES, minor/major allele; P, p value in Cochran-Armitage trend test; LOCATION, genomic location of the marker – for markers of regions identified by GWAS references are also given.

NR	CHR	SNP	ALLELES	**P**	LOCATION
1.	1p13.2	rs3811021	G/A	**0.0333**	*PTPN22*
2.	1p13.2	rs2476599	A/G	**0.0027**	*PTPN22*
3.	1p13.2	rs2476601	A/G	**0.0687**	*PTPN22*
4.	1p13.2	rs2488457	G/C	**0.3392**	*PTPN22* promoter region
5.	1q32.1	rs1800896	G/A	**0.0997**	*IL10* promoter region
6.	2p25.3	rs11211645	G/A	**0.0127**	*TPO* promoter region
7.	2p25.3	rs961028	G/A	**0.1837**	*TPO*
8.	2p25.3	rs2276704	A/G	**0.2864**	*TPO*
9.	2q24.2	rs1990760	G/A	**0.0281**	*IFIH1*
10.	2q24.2	rs3747517	A/G	**0.0517**	*IFIH1*
11.	2q33.2	rs16840252	A/G	**0.3744**	*CTLA4* promoter region
12.	2q33.2	rs231775	G/A	**0.0586**	*CTLA4*
13.	2q33.2	rs3087243	A/G	**0.2795**	3’ to *CTLA4*
14.	6p21.33	rs1800629	A/G	**0.3718**	*TNF* promoter region
15.	7p15.3	rs1800795	G/C	**0.5504**	*IL6* promoter region
16.	8q24.22	rs180223	A/C	**0.1370**	*TG*
17.	8q24.22	rs2069550	A/G	**0.2235**	*TG*
18.	8q24.22	rs16905194	T/A	**0.4267**	*ZFAT*
19.	10q11.21	rs1800863	C/G	**0.3769**	*RET*

20.	10q21.2	rs6479778	A/G	**0.7426**	*ARID5B*
21.	12q21.33	rs566806	A/G	**0.4658**	*DCN* [1]
22.	12q21.33	rs6538281	A/G	**0.7510**	intergenic [1]
23.	13q33.3	rs9558786	A/C	**0.4799**	*ARGLU1* [1]
24.	13q33.3	rs2769917	A/C	**0.0449**	intergenic [1]
25.	13q33.3	rs9520584	A/G	**0.2901**	*FAM155A* [1]
26.	14q24.2	rs7140236	G/A	**0.1613**	*TTC9* [2]
27.	14q24.2	rs11158882	G/C	**0.8195**	*MAP3K9* [2]
28.	14q24.2	rs2074953	G/A	**0.0047**	*RGS6* [2]
29.	14q24.2	rs165933	A/G	**0.0571**	*PSEN1* [2]
30.	20q12	rs3577	A/G	**0.5469**	*MAFB* [3]
31.	20q12	rs753381	A/G	**0.1829**	*PLCG1* [3]
32.	20q12	rs6072392	G/A	**0.1403**	*CHD6* [3]
33.	20q13.12	rs4810485	A/C	**0.4314**	*CD40*
34.	Xp11.23	rs3761549	A/G	**0.5277**	*FOXP3*
35.	Xq22.1	rs5966709	A/C	**0.0023**	*TNMD* [1]
36.	Xq22.1	rs2027829	A/C	**0.0861**	*SYTL4* [1]
37.	Xq22.1	rs5921679	A/G	**0.0375**	*NOX1* [1]

1 Tomer Y, Barbesino G, Greenberg DA, Concepcion E & Davies TF (1999) Mapping the major susceptibility loci for familial Graves' and Hashimoto's diseases: Evidence for genetic heterogeneity and gene interactions. *J. Clin. Endocrinol. Metab.* **84**, 4656–4664.
2 Tomer Y, Ban Y, Concepcion E, Barbesino G, Villanueva R, Greenberg D a & Davies TF (2003) Common and unique susceptibility loci in Graves and Hashimoto diseases: results of whole-genome screening in a data set of 102 multiplex families. *Am. J. Hum. Genet.* **73**, 736–747.
3 Tomer Y, Barbesino G, Greenberg DA & Concepcion E (1998) A New Graves Disease – Susceptibility Locus Maps To Chromosome 20q11 . 2. *New York*, 1749–1756.
